# Supplementary material for: TRPA1 Covalent Ligand JT010 Modifies T Lymphocyte Activation
Source: Biomolecules. 2024 May 28;14(6):632. doi: 10.3390/biom14060632 (PMC11202300; doi:10.3390/biom14060632)
Supplement: Supplementary file 1 [file biomolecules-14-00632-s001.zip › biomolecules-3012327-supplementary.pdf]

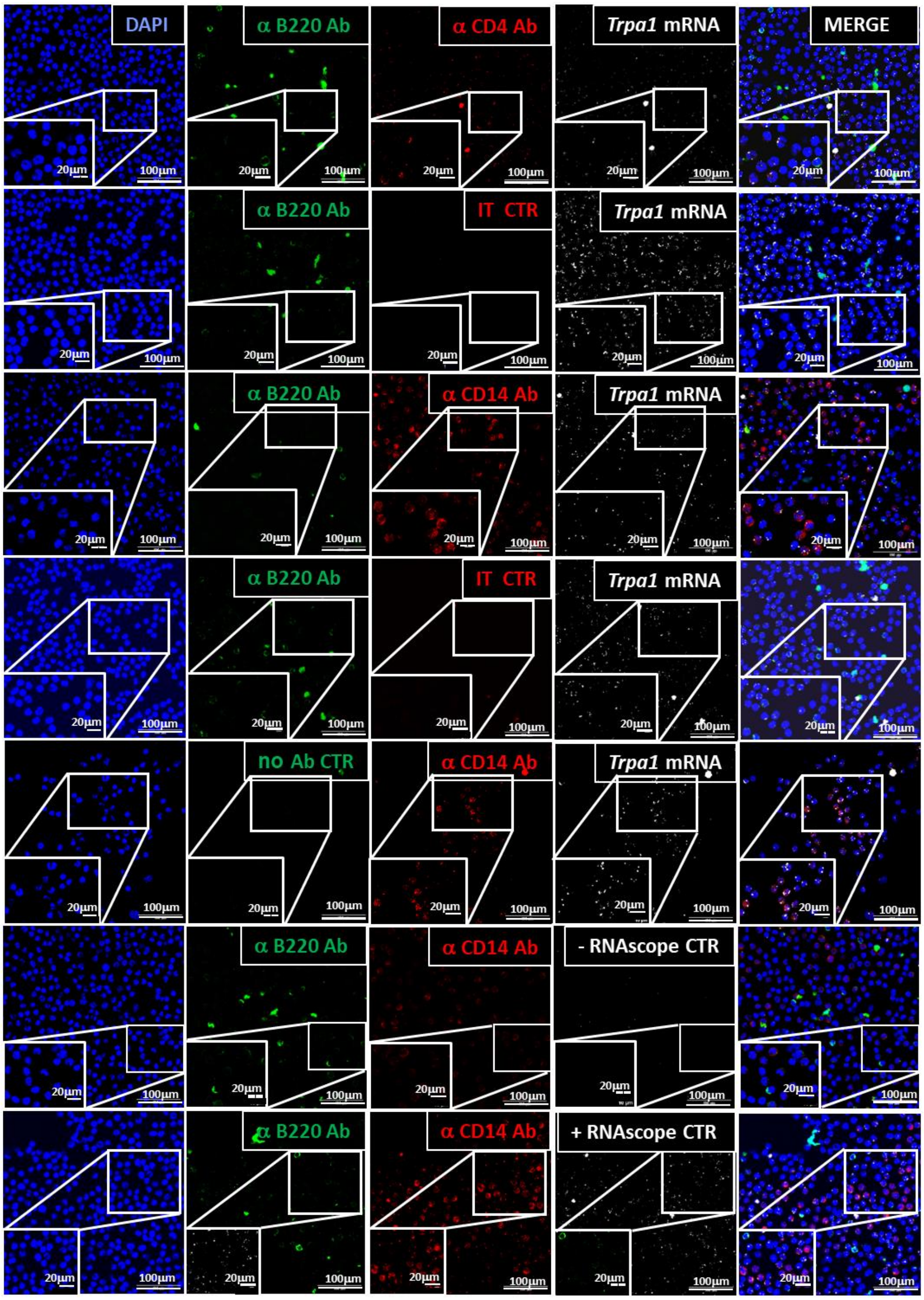

Figure S1. *Trpa1* mRNA co-localization with CD4, CD14 and B220 CD markers. Representative RNAscope results compared to RNAscope positive (+) and negative (–) controls. White RNAscope® ISH signals in the images indicated one of the following: *Trpa1* mRNA, RNA polymerase II subunit A mRNA (+ RNAscope CTR), or bacterial dihydrodipicolinate reductase mRNA (- RNAscope CTR). Red immunofluorescent signals indicated in the images one of the following: anti-CD4 antibody (αCD4 Ab), anti- CD14 antibody (αCD14 Ab), or isotype control antibody-labelled (IT CTR) cells. Green immunofluorescent signals indicate in the images the anti-B220 antibody-labelled cells (αB220 Ab). Nuclear counterstaining was performed with 4′,6- diamidino-2-phenylindole (DAPI, blue).

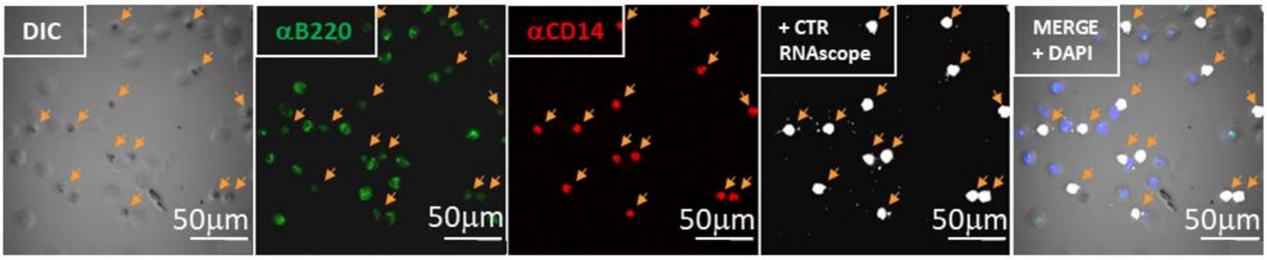

Figure S2. Representative dual RNAscope® ISH-IF technique results with artifacts. Cell morphology by differential interference contrast (DIC) after immunofluorescent surface labelling of 97% viable cells followed by formalin fixation and then RNAscope mRNA detection procedure. White RNAscope® ISH signals indicated the RNA polymerase II subunit A mRNA (+ CTR RNAscope). Red immunofluorescent signals indicated the anti- CD14 antibody labeling. Green immunofluorescent signals indicated the anti-B220 antibody-labelled cells. Nuclear counterstaining was performed with 4′,6- diamidino-2-phenylindole (DAPI, blue).

This Figure demonstrates that cells with darker DIC phenotype shows strong staining with antibodies (here anti-CD14 antibody) and also strikingly strong labeling for the houskeeping mRNA probe developed by RNAscope technic, indicating non-specific labeling of the cells.

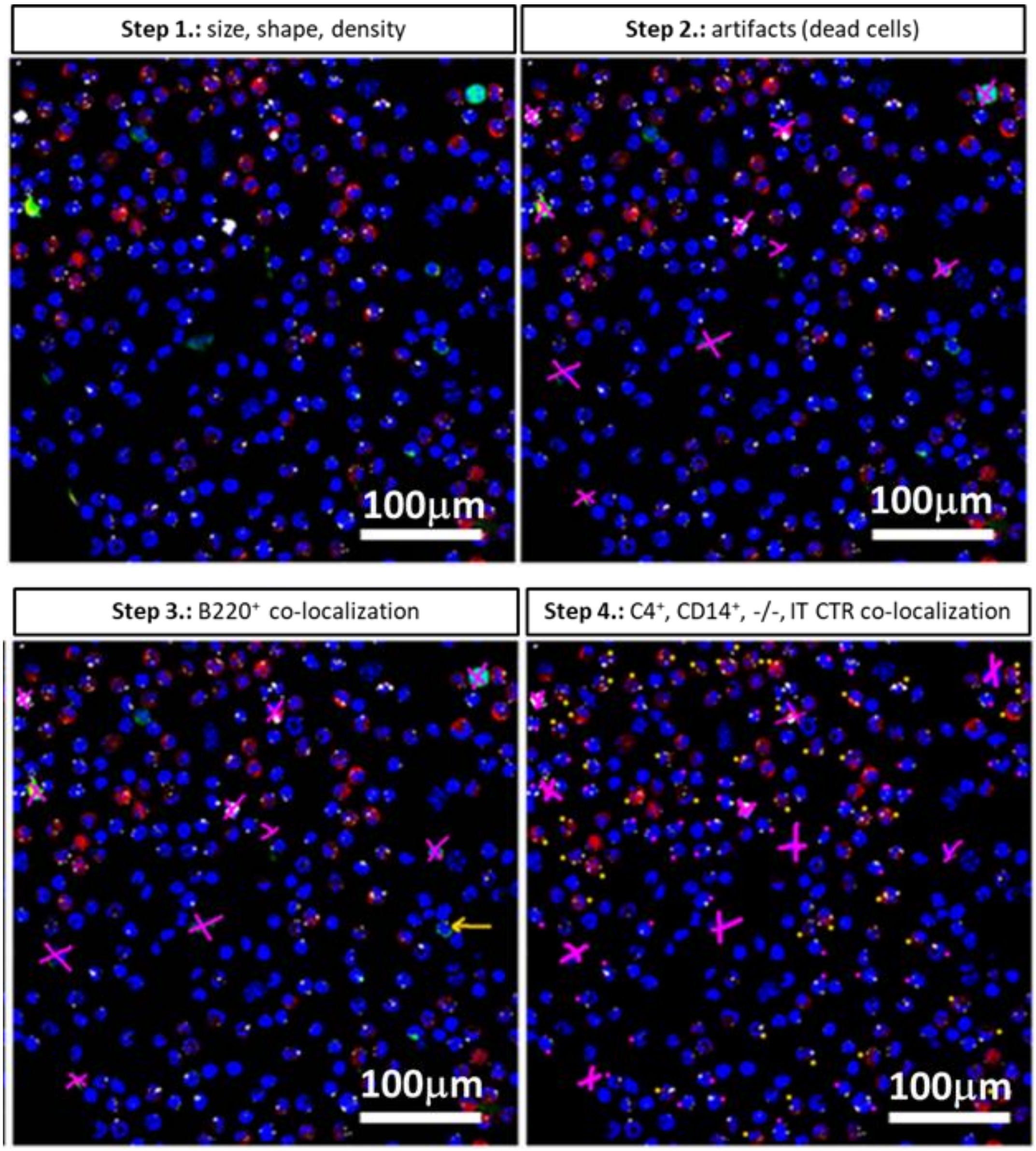

Figure S3. Quantification *Trpa1* mRNA. Scheme of quantification (CellProfiler pipeline) steps in order: Step 1: binarization of the image by specifying the required smoothing and threshold parameters, filter binary objects based on size, shape and density; Step 2: deletion of objects marking dead cells (crossed with lilac in the second panel); Step 3: counting of B220+ cells (green in the Figure) that co-localized with mRNA (white in the Figure), Step 4: counting of C4+,CD14+, and isotype control antibody-labelled (IT CTR) cells (red in the Figure) and non-CD marker antibody-labelled cells (-/-), that co-localized with mRNA (white in the Figure).

See detailed method for dual RNAscope® ISH-IF technique in chapter 2.2 and 2.3. White RNAscope® ISH signals in the images indicated one of the following: *Trpa1* mRNA, *RNA polymerase II subunit A* mRNA (+ RNAscope CTR), and *bacterial dihydrodipicolinate reductase* mRNA (- RNAscope CTR).
Red immunofluorescent signals indicate in the images one of the following: anti-CD4 antibody (CD4+), anti-CD14 antibody (C14+), or isotype control antibody-labelled (IT CTR) cells. Green immunofluorescent signals indicate in the images the anti-B220 antibody-labelled cells (B220+).
